# Combination Kinase Inhibitor Treatment Suppresses Rift Valley Fever Virus Replication

**DOI:** 10.3390/v10040191

**Published:** 2018-04-13

**Authors:** Todd M. Bell, Virginia Espina, Lindsay Lundberg, Chelsea Pinkham, Ashwini Brahms, Brian D. Carey, Shih-Chao Lin, Bibha Dahal, Caitlin Woodson, Cynthia de la Fuente, Lance A. Liotta, Charles L. Bailey, Kylene Kehn-Hall

**Affiliations:** 1 National Center for Biodefense and Infectious Diseases, School of Systems Biology, George Mason University, Manassas, VA 20110, USA; tbell11@masonlive.gmu.edu (T.M.B.); lhill10@masonlive.gmu.edu (L.L.); cpinkham@masonlive.gmu.edu (C.P.); abenedi3@masonlive.gmu.edu (A.B.); bcarey4@masonlive.gmu.edu (B.D.C.); slin20@gmu.edu (S.-C.L.); bdahal@masonlive.gmu.edu (B.D.); cwoodso2@gmu.edu (C.W.); cdelafue@gmu.edu (C.d.l.F.); cbailey2@gmu.edu (C.L.B.); 2 Center for Applied Proteomics and Molecular Medicine, School of Systems Biology, George Mason University, Manassas, VA 20110, USA; vespina@gmu.edu (V.E.); lliotta@gmu.edu (L.A.L.)

**Keywords:** Rift Valley fever virus, kinase, inhibitor, p38 MAPK, ERK, p70 S6K, p90RSK, mTOR, rapamycin, translation

## Abstract

Viruses must parasitize host cell translational machinery in order to make proteins for viral progeny. In this study, we sought to use this signal transduction conduit against them by inhibiting multiple kinases that influence translation. Previous work indicated that several kinases involved in translation, including p70 S6K, p90RSK, ERK, and p38 MAPK, are phosphorylated following Rift Valley fever virus (RVFV) infection. Furthermore, inhibiting p70 S6K through treatment with the FDA approved drug rapamycin prevents RVFV pathogenesis in a mouse model of infection. We hypothesized that inhibiting either p70 S6K, p90RSK, or p90RSK’s upstream kinases, ERK and p38 MAPK, would decrease translation and subsequent viral replication. Treatment with the p70 S6K inhibitor PF-4708671 resulted in decreased phosphorylation of translational proteins and reduced RVFV titers. In contrast, treatment with the p90RSK inhibitor BI-D1870, p38MAPK inhibitor SB203580, or the ERK inhibitor PD0325901 alone had minimal influence on RVFV titers. The combination of PF-4708671 and BI-D1870 treatment resulted in robust inhibition of RVFV replication. Likewise, a synergistic inhibition of RVFV replication was observed with p38MAPK inhibitor SB203580 or the ERK inhibitor PD0325901 combined with rapamycin treatment. These findings serve as a proof of concept regarding combination kinase inhibitor treatment for RVFV infection.

## 1. Introduction

Rift Valley fever virus (RVFV), an emerging pathogen and public health threat, was identified as the causative agent of an outbreak of viral hemorrhagic fever in Kenya in 1930 [1,2]. RVFV was initially only found in Sub-Saharan Africa, but since its discovery, it has spanned the African mainland, crossed over to Madagascar, and moved into the Arabian Peninsula [3,4,5,6,7,8,9,10,11]. The footprint of RVFV mosquito vectors has expanded, and this, in combination with the ubiquity of competent mammalian hosts [4,12,13,14,15,16], makes the likelihood of this virus spreading globally a distinct possibility [17,18]. The alarming escape of other arthropod-borne viruses (West Nile virus, chikungunya virus, Zika virus) from their ecological niches, into and throughout the Americas [19,20,21,22,23,24] gives us a potential harbinger of future events. Based on these concerning trends, governments and public health authorities across the globe have identified a need for FDA approved vaccines and treatments for RVFV [1,25].

Viruses encode few proteins and as such utilize host cell signaling pathways to meet their replication and propagation needs. RVFV, for which we have limited molecular pathogenesis knowledge, is no exception. In order to understand how to attack viruses therapeutically, a detailed understanding of how they manipulate host cell machineries is essential. Previous work indicated that several kinases involved in translation were upregulated following RVFV infection [26]. Specifically, reverse phase protein array analysis showed that phosphorylation of 70-kDa S6 kinase (p70 S6K), 90-kDa ribosomal S6 kinase (p90RSK), extracellular signal-regulated kinases (ERK), and p38 mitogen-activated protein kinases (p38 MAPK) was increased following RVFV infection [26,27]. The S6 ribosomal protein, a substrate for both p70 S6K and p90RSK, and eIF4G, a protein important in initiation of cap-dependent translation, are also phosphorylated following RVFV infection [27]. p38 MAPK influences translation through phosphorylation of mitogen-activated protein kinase-interacting protein (MNK), which phosphorylates eIF4E [28,29]. In addition, it is an upstream regulator of p90RSK as inhibition of p38 MAPK through SB203580 treatment resulted in decreased phosphorylation of p90RSK at Ser 380 [30]. p38 MAPK, through the intermediates MAPK-activated kinase 2 (MK2) and MK3, can activate p90RSK in certain situations, such as viral infection [30,31]. ERK once activated by phosphorylation of Thr 202/Tyr 204 in the activation loop can phosphorylate p90RSK promoting cap-dependent translation [32,33]. Because kinases are the levers by which signal transduction pathways are turned on and off [34,35], modulation of these kinases allows the determination of their importance in the viral life cycle. An enticing reason to explore kinase modulation following infection is the large number of kinase inhibitors in use for cancer treatment [36,37], which makes for a large potential armamentarium for future use against viruses. Inhibition of translation through the use of the FDA approved mTOR inhibitor, rapamycin, protects mice from RVFV induced pathogenesis [27], providing evidence that inhibition of translation is a viable therapeutic target. This inhibition is at least partially mediated through p70 S6 kinase, a kinase integral to the successful activation of the translation pathway. Rapamycin treatment decreased RVFV N protein (NP) production and RVFV replication in vitro. However, rapamycin has several additional effects aside from modulation of the p70 S6K pathway; therefore we wanted to test additional inhibitors of the translation pathway including a direct p70 S6K inhibitor. In this study the ability of the p70 S6K inhibitor PF-4708671, p90RSK specific inhibitor BI-D1870, the p38MAPK inhibitor SB203580, and the ERK inhibitor PD0325901 to alter RVFV replication in hepatocytes was determined. Combination kinase inhibitor treatments were also analyzed to test the hypothesis that targeting more than one kinase would results in more potent inhibition of RVFV replication. 

## 2. Materials and Methods

### 2.1. Cell Culture

H2.35 (ATCC, CRL-1995) cells were maintained in Dulbecco’s low glucose modified minimum essential medium + GlutaMAX with 200 nM dexamethasone and 4.0% heat-inactivated fetal bovine serum (FBS). Vero cells (ATCC, Manassas, VA, USA, CCL-81) were maintained in Dulbecco’s modified minimum essential medium (DMEM) supplemented with 10% FBS, 1% l-glutamine, and 1% penicillin/streptomycin. H2.35 cells were maintained at 33 °C with 10% CO_2_ while Vero cells were maintained at 37 °C with 5% CO_2_.

### 2.2. Viral Infections

RVFV vaccine strain MP-12 virus was rescued and titered as previously described [38,39,40]. For viral infections of H2.35 cells (mouse hepatocytes), cells were cultured in 12 well plates and grown to 80–90% confluency. Cells were serum starved for 72 h to synchronize cells in G0/G1 phase [41]. The cells were infected with RVFV MP12 at a multiplicity of infection (MOI) of 5. Cells were incubated for 1 h at 33 °C and at 10% CO_2,_ with rocking of cells every 15 min. Infectious media was removed, cells washed once with phosphate buffered saline (PBS) without Ca^2+^ and Mg^2+^, and complete media added. The cells were left to incubate and collected using the appropriate method for downstream applications at the specified time points.

### 2.3. Western Blot

Protein lysates were collected and analyzed by western blot as previously described [42]. In brief, primary antibodies against p90RSK (Ser380) (Cell Signaling Technology, Danvers, MA, USA, 9341), p70 S6 Kinase (Thr389) (Cell Signaling Technology 9205), S6 ribosomal protein (Ser235/236) (Cell Signaling Technology, 4856), eIF4G (Ser1108) (Cell Signaling Technology, 2441), RVFV MP12 Antibody (IBT Bioservices, Rockville, MD, USA, 04-0001), or HRP-conjugated actin (Abcam, ab49900) were diluted 1:1000 in 5% bovine serum albumin in 1× TBS with 0.1% Tween-20 solution followed by the addition of the appropriate secondary antibody. The western blots were visualized by chemiluminescence using SuperSignal West Femto Maximum Sensitivity Substrate kit (ThermoScientific, Waltham, MA, USA) and a Bio Rad Molecular Imager ChemiDoc XRS system (Bio-Rad, Hercules, CA, USA) [41,42].

### 2.4. Plaque Assay

Extracellular supernatants were collected at the indicated time points and stored at −80 °C. Viral titers were determined by plaque assay using Vero cells as previously described [43].

### 2.5. Cytotoxic Concentration 50 (CC_50_) Assays

Cells were plated in a white-walled 96-well plate and allowed to incubate at 33 °C and 10% CO_2_ overnight. H2.35 cells were treated with drug concentrations starting at 100 micromolar (µM) with 1:2 serial dilutions down to 1.56 µM, and drugs were incubated with cells for 24 h. Drugs were added either alone or in combination, in equal concentrations per well, as needed to determine toxicity of each drug or drug combination. At 24 h, all cells were analyzed using Cell Titer-Glo Cell Luminescent Viability Assay (Promega, G7570, Madison, WI, USA) according to vendor’s instructions. This assay measures relative ATP levels. Briefly, an equal volume of room temperature media and Cell Titer-Glo reagent were added to the cells. The plate was shaken for 2 min on an orbital shaker followed by a 10 min room temperature incubation. Viability was detected via luminescence detection using the DTX 880 multimode detector (Beckman Coulter, Brea, CA, USA) and percent viability was calculated relative to the DMSO control.

### 2.6. Effective Concentration 50 (EC_50_) Assays

Cells were plated in a white-walled 96-well plate and allowed to incubate at 33 °C and 10% CO_2_ overnight. H2.35 cells were pre-treated for 1 h with various drug concentrations (1:2 serial dilutions). Drugs were added either alone or in combination, in equal concentrations per well, as needed to determine efficacy of each drug or drug combination. Cells were then infected with RVFV MP12 ∆NSs-Luc (MP12 lacking the NSs gene and replaced by a gene encoding *Renilla* luciferase) at a MOI of 0.1. Cells were incubated for 1 h at 33 °C and at 10% CO_2_. Infectious media was removed, cells washed once with PBS without Ca^2+^ and Mg^2+^, and drug was re-applied. Cells were analyzed at 18 hpi using Renilla-Glo^®^ Luciferase Assay System (Promega) according to vendor’s instructions. Briefly, an equal volume of room temperature media and Renilla-Glo reagent was added to the cells. The plate was incubated for at least 10 min at room temperature. Luminescence was detected via luminescence detection using the DTX 880 multimode detector (Beckman Coulter) and percent luminescence was calculated relative to the DMSO control.

### 2.7. Treatments

Rapamycin was obtained from LC Laboratories (Cat No. R-5000), the ERK inhibitor PD0325901 was obtained from MedChem Express (Cat No. HY-10254), the p38MAPK inhibitor SB203580 was from Selleckchem (Cat. No. S1076), the p90RSK inhibitor BI-D1870 was purchased from MedChem Express (Cat. No. HY-10510) and the p70 S6K inhibitor PF-4708671 was from EMD/Millipore (Cat. No. 559273). H2.35 cells were treated for 1 h with DMSO or the inhibitor(s) of choice for the given experiment. After pretreatment, drug was removed, and cells were infected for 1 h. After washing out virus inoculum, cells were cultured in complete media with drug and cells collected at the respective assay time points. 

### 2.8. Statistics

Statistical analyses, unpaired, two-tailed student *t*-tests and Extra sum-of-squares *F* test, were performed using Graphpad Prism software (version 7, La Jolla, CA, USA).

## 3. Results

### 3.1. The p70 S6K Inhibitor PF-4708671 Alone or in Combination with the p90RSK Inhibitor BI-D1870 Decreases RVFV Replication In Vitro

**Figure 1:**
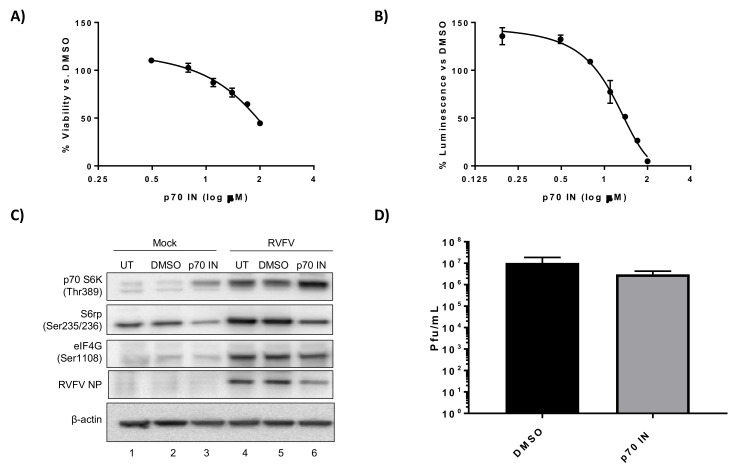
PF-4708671, a p70 S6K inhibitor, decreases RVFV N protein (NP) translation and replication. (**A**) H2.35 cells were treated with PF-4708671 p70 S6K inhibitor (p70 IN) and cell viability analyzed at 24 h using Cell Titer-Glo. Percent viability was calculated relative to the DMSO control. The mean and SD (N = 3) are plotted; (**B**) H2.35 cells were pre-treated for 1 h, followed by a 1 h infection with RVFV MP12 ∆NSs-Luc (MOI of 0.1). Cells were analyzed at 18 hpi using the Renilla Glo Assay. Luminescence is presented relative to the DMSO control. The mean and SD (N = 3) are plotted; (**C**) H2.35 cells were serum starved for 72 h and then were pre-treated with either DMSO or p70 IN (10 µM). Cells were infected with MP12 (MOI 5) for one h, followed by removal of viral inoculum, and addition of growth medium containing DMSO or p70 IN (10 µM). At 18 hpi, cell lysates were collected for western blot analysis. UT = untreated. Images are representative of biological replicates; (**D**) H2.35 cells were treated and infected as described in (**C**). At 18 hpi, supernatants were collected for standard plaque assay. The mean and SD (N = 3) are plotted.

p70 S6K and p90RSK are both capable of activating the S6 ribosomal protein through phosphorylation of Ser 235/236 and thus facilitating translation activation [44,45]. Therefore inhibitors targeting p70 S6K and p90RSK either alone or in combination were evaluated to determine if they influenced RVFV replication in mouse hepatocytes. We chose the p70 S6K inhibitor PF-4708671 for this analysis, as this compound inhibits p70 S6K activity with high specificity [46,47]. Cell cytotoxicity 50 (CC_50_) and effective concentration 50 (EC_50_) assays were performed to rule out mouse hepatocyte cell toxicity and determine an appropriate dose to use in our in vitro efficacy studies. H2.35 BALB/c hepatocytes were somewhat tolerant of the p70 S6K inhibitor PF-4708671, with a CC_50_ of over 50 µM (Figure 1A, Table 1). PF-4708671 displayed an EC_50_ of 17 µM against RVFV MP12-luc (Figure 1B, Table 1). Analysis of cell signaling proteins demonstrated increased phosphorylation of p70 S6K (Thr 389), and downstream proteins S6 ribosomal protein (Ser 235/235) and eIF4G (Ser 1108) following RVFV infection, in agreement with our previous results [27] (Figure 1C, compare lanes 1 and 2 to lanes 4 and 5). Treatment with PF-4708671 resulted in decreased phosphorylation of S6 ribosomal protein (Ser 235/235), but not p70 S6K (Thr 389) and eIF4G (Ser 1108) (Figure 1C, lanes 3 and 6). A decrease in p70 S6K Thr389 phosphorylation is not surprising, given that this is not an autophosphorylation site and rather Thr389 is phosphorylated by mTOR [48]. Additionally, the levels of RVFV NP were decreased following PF-4708671 treatment (Figure 1C, lane 6), consistent with inhibition of viral translation. A reduction in viral titers was also observed, although not statistically significant (Figure 1D). These data confirm that p70S6K inhibition suppresses RVFV replication. 

**Figure 2:**
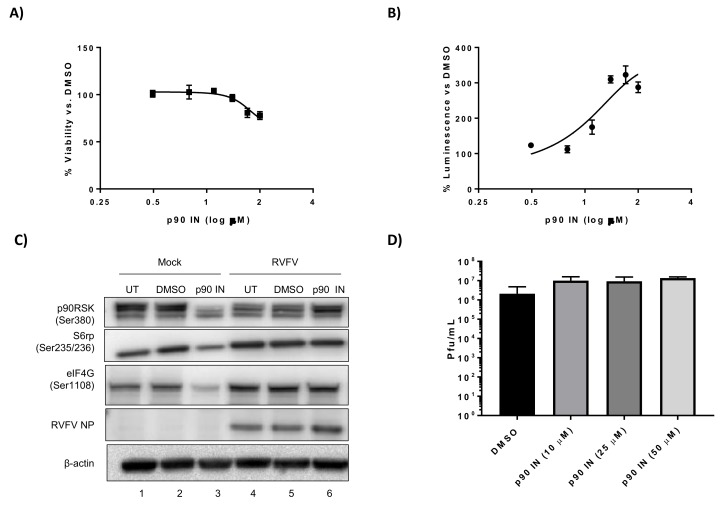
BI-D1870, a p90RSK inhibitor, increases RVFV replication. (**A**) H2.35 cells were treated with BI-D1870 p90RSK inhibitor (p90 IN) and cell viability analyzed at 24 h using Cell Titer-Glo. Percent viability was calculated relative to the DMSO control. The mean and SD (N = 3) are plotted; (**B**) H2.35 cells were pre-treated for 1 h, followed by a 1 h infection with RVFV MP12 ∆NSs-Luc (MOI of 0.1). Cells were analyzed at 18 hpi using the Renilla Glo Assay. Luminescence is presented relative to the DMSO control. The mean and SD (N = 3) are plotted; (**C**) H2.35 cells were serum starved for 72 h and then were pre-treated with either DMSO or p90 IN (10 µM). Cells were infected with MP12 (MOI 5) for one hour, followed by removal of viral inoculum, and addition of growth medium containing DMSO or p90 IN (10 µM). At 18 hpi, cell lysates were collected for western blot analysis. UT = untreated. Images are representative of biological replicates; (**D**) H2.35 cells were treated and infected as described in (**C**). At 18 hpi, supernatants were collected for standard plaque assay. The mean and SD (N = 3) are plotted.

The p90RSK inhibitor, BI-D1870 [49], has been used in chemotherapy studies showing specific inhibition of p90RSK activity [50], therefore we used this drug to inhibit p90RSK in the context of RVFV infection. H2.35 cells were very tolerant of BI-D1870 treatment, with only a ~20% decrease in viability observed at the highest concentration tested (100 µM) (Figure 2A). In contrast, when we attempted to determine the EC_50_ of the p90RSK inhibitor BI-D1870, it was shown that as the drug concentration increased, viral replication increased as well (Figure 2B). Inhibition of phosphorylation of p90RSK, S6 ribosomal protein, as well as eIF4G decreased following drug treatment in mock infected samples (Figure 2C, Lane 3). Surprisingly, drug treatment in the context of MP12 infection resulted in no apparent change in phosphorylation of p90RSK, S6 ribosomal protein and eIF4G, but modestly increased the amount of RVFV NP (Figure 2C, Lane 6). The level of infectious viral titer increased by approximately 0.5 log at all drug concentrations tested (Figure 2D). These results indicate that infected cells are more resistant to p90RSK inhibition and/or BI-D1870, a p90RSK inhibitor, is a viral agonist, not antagonist as it relates to infection of mouse hepatocytes. 

**Figure 3:**
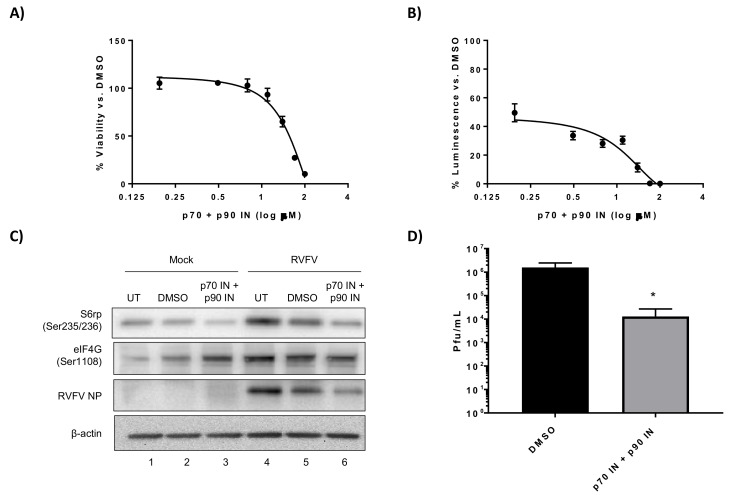
Combination kinase inhibitor treatment consisting of p70 S6K and p90RSK inhibitors decreases RVFV replication and NP translation. (**A**) H2.35 cells were treated with a combination of PF-4708671 p70 S6K inhibitor (p70 IN) and BI-D1870 p90RSK inhibitor (p90 IN) and cell viability analyzed at 24 h using Cell Titer-Glo. Percent viability was calculated relative to the DMSO control. The mean and SD (N = 3) are plotted; (**B**) H2.35 cells were pre-treated for 1 h, followed by a 1 h infection with RVFV MP12 ∆NSs-Luc (MOI of 0.1). Cells were analyzed at 18 hpi using the Renilla Glo Assay. The mean and SD (N = 3) are plotted; (**C**) H2.35 cells were serum starved for 72 h and then were pre-treated with either DMSO or p70 IN/p90 IN combination (10 µM). Cells were infected with MP12 (MOI 5) for one hour, followed by removal of viral inoculum, and addition of growth medium containing DMSO or p70 IN/p90 IN combination (10 µM). At 18 hpi, cell lysates were collected for western blot analysis. UT = untreated. Images are representative of biological replicates; (**D**) H2.35 cells were treated and infected as described in (**C**). At 18 hpi, supernatants were collected for standard plaque assay. The mean and SD (N = 3) are plotted. * *p*-value ≤ 0.05.

Kinase inhibitors in chemotherapy treatments can be used alone or in combination, and we wanted to determine if a synergistic effect could be achieved by using two kinase inhibitors simultaneously in RVFV infected cells. Even though p90RSK on its own resulted in more viral replication, we aimed to determine if a p70 S6K and p90RSK inhibitor combination could mitigate these kinases’ redundancy in terms of S6 ribosomal protein phosphorylation resulting in a more global shutdown of phosphorylation leading to a decrease in RVFV protein production and replication. CC_50_ and EC_50_ assays were performed on the combination treatments, as it is likely that a combination of the two inhibitors would be less tolerated than single treatment regimens. H2.35 cell viability was more greatly affected with the drug combination of the p90RSK inhibitor BI-D1870 and the p70 S6K inhibitor PF-4708671, with a calculated CC_50_ of 62 µM (Figure 3A, Table 1). This inhibitor combination was very effective at suppressing RVFV with ~70% inhibition observed at 6.25 µM (Figure 3B), whereas either treatment alone did not inhibit RVFV at this concentration (Figure 1B and Figure 2B). Protein phosphorylation (Figure 3C) and infectious titers (Figure 3D) were evaluated in the presence of 10 µM of the drugs, a concentration that has minimal effect on cell viability. Phosphorylation of S6 ribosomal protein decreased following drug treatment (Figure 3C, Lane 6). Additionally, the levels of RVFV NP and RVFV titers (Figure 3C,D) were significantly decreased. These results demonstrate that using the p70 S6 kinase inhibitor PF-4708671 in combination with the p90RSK inhibitor BI-D1870 resulted in a significant decrease in RVFV replication and viral protein production. 

### 3.2. The p38 MAPK Inhibitor SB203580 and ERK Inhibitor PD0325901 have Minimal Effect on RVFV Replication In Vitro

**Figure 4:**
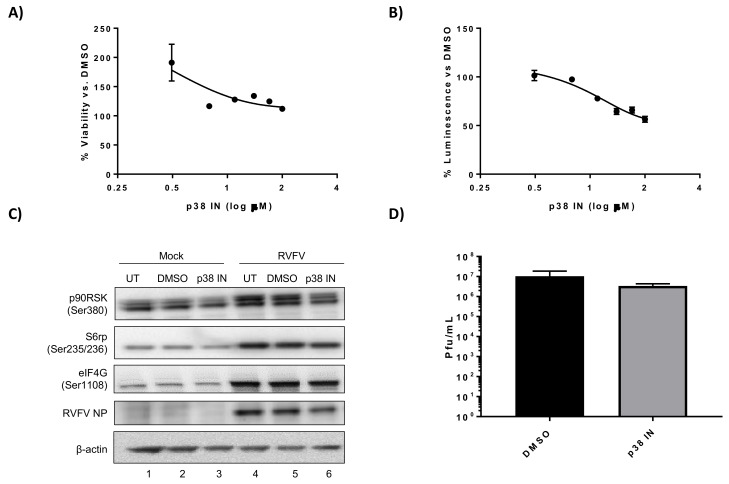
SB203580, a p38 MAPK inhibitor, has a minimal effect on RVFV replication. (**A**) H2.35 cells were treated with SB203580 p38MAPK inhibitor (p38 IN) and cell viability analyzed at 24 h using Cell Titer-Glo. Percent viability was calculated relative to the DMSO control. The mean and SD (N = 3) are plotted; (**B**) H2.35 cells were pre-treated for 1 h, followed by a 1 h infection with RVFV MP12 ∆NSs-Luc (MOI of 0.1). Cells were analyzed at 18 hpi using the Renilla Glo Assay. Luminescence is presented relative to the DMSO control. The mean and SD (N = 3) are plotted; (**C**) H2.35 cells were serum starved for 72 h and then were pre-treated with either DMSO or 10 µM p38 IN. Cells were infected with MP12 (MOI 5) for one hour, followed by removal of viral inoculum, and addition of growth medium containing DMSO or 10 µM p38 IN. At 18 hpi, cell lysates were collected for western blot analysis. UT = untreated. Images are representative of biological replicates; (**D**) H2.35 cells were treated and infected as described in (**C**). At 18 hpi, supernatants were collected for standard plaque assay. The mean and SD (N = 3) are plotted.

**Figure 5:**
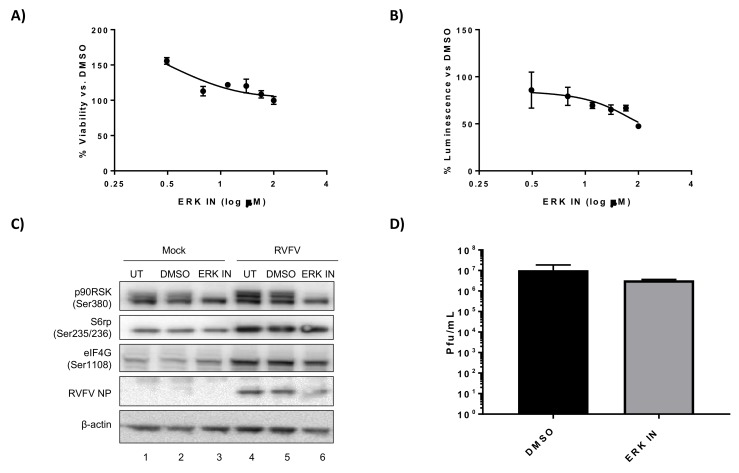
PD0325901, an ERK inhibitor, has a minimal effect on RVFV replication. (**A**) H2.35 cells were treated with PD0325901 ERK inhibitor (ERK IN) and cell viability analyzed at 24 h using Cell Titer-Glo. Percent viability was calculated relative to the DMSO control. The mean and SD (N = 3) are plotted; (**B**) H2.35 cells were pre-treated for 1 h, followed by a 1 h infection with RVFV MP12 ∆NSs-Luc (MOI of 0.1). Cells were analyzed at 18 hpi using the Renilla Glo Assay. Luminescence is presented relative to the DMSO control. The mean and SD (N = 3) are plotted; (**C**) H2.35 cells were serum starved for 72 h and then were pre-treated with either DMSO or 10 µM ERK IN. Cells were infected with MP12 (MOI 5) for one hour, followed by removal of viral inoculum, and addition of growth medium containing DMSO or 10 µM ERK IN. At 18 hpi, cell lysates were collected for western blot analysis. UT = untreated. Images are representative of biological replicates; (**D**) H2.35 cells were treated and infected as described in (**C**). At 18 hpi, supernatants were collected for standard plaque assay. The mean and SD (N = 3) are plotted.

To further explore kinases involved in regulation of the translation pathway, we evaluated the importance of p38 MAPK and ERK, both of which can influence phosphorylation of p90RSK [30,32]. To this end, the p38 MAPK inhibitor SB203580 and the ERK inhibitor PD0325901 were evaluated. SB203580 was the first identified p38 MAPK inhibitor and prevents p38 MAPK activity through competitively binding to the ATP pocket [51]. PD0325901 binds to and inhibits MEK, preventing phosphorylation and activation of ERK [52]. H2.35 cells were very tolerant of the p38 MAPK inhibitor SB203580, with no appreciable cell toxicity observed even at 100 µM (Figure 4A). Likewise, only a ~20% decrease in H2.35 cell viability was observed at the highest concentration of the ERK inhibitor PD0325901 tested (100 µM) (Figure 5A). While SB203580 and PD0325901 both inhibited RVFV, they weren’t very potent against RVFV MP12-luc as even 100 µM only resulted in 45% inhibition (Figure 4B) and 52% inhibition (Figure 5B), respectively. Inhibition of p38 MAPK resulted in minimal reduction of p90RSK, whereas inhibition of ERK resulted in an appreciable decreased in p90RSK Ser380 phosphorylation (Figure 4B and Figure 5B). Additionally, inhibition of p38 MAPK or ERK resulted in only minimal inhibition of S6 ribosomal protein and eIF4G phosphorylation, RVFV NP levels and viral titers (Figure 4C,D and Figure 5C,D). These results demonstrate that the p38 MAPK inhibitor SB203580 and the ERK inhibitor PD0325901 have limited effect on RVFV replication when used alone. 

### 3.3. The p38 MAPK Inhibitor SB203580 or the ERK Inhibitor PD0325901 in Combination with Rapamycin Significantly Decreases RVFV Replication In Vitro

**Figure 6:**
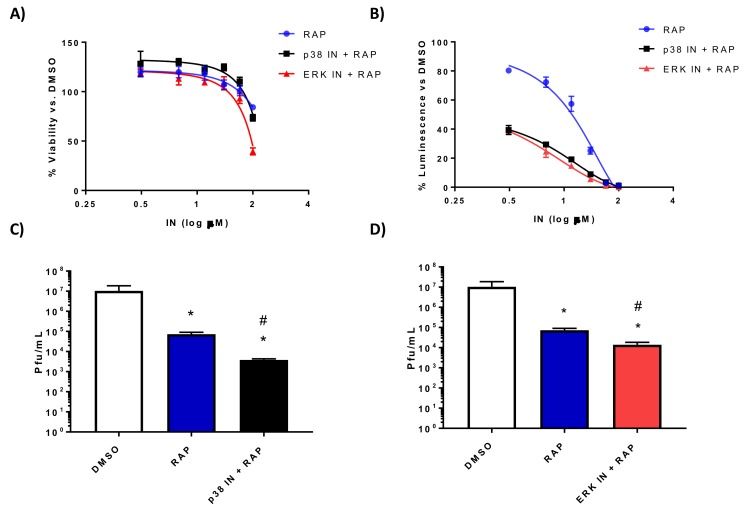
Rapamycin in combination with the p38 MAPK inhibitor SB203580 or the ERK inhibitor PD0325901 significantly decreases RVFV replication. (**A**) H2.35 cells were treated with rapamycin (RAP) alone or in combination with SB203580 p38MAPK inhibitor (p38 IN) or PD0325901 ERK inhibitor (ERK IN) and cell viability analyzed at 24 h using Cell Titer-Glo. Percent viability was calculated relative to the DMSO control. The mean and SD (N = 3) are plotted; (**B**) H2.35 cells were pre-treated for 1 h, followed by a 1 h infection with RVFV MP12 ∆NSs-Luc (MOI of 0.1). Cells were analyzed at 18 hpi using the Renilla Glo Assay. Luminescence is presented relative to the DMSO control. The mean and SD (N = 3) are plotted; (**C**,**D**) H2.35 cells were serum starved for 72 h and then were pre-treated with either 10 µM RAP, RAP + p38 IN, or RAP +ERK IN. Cells were infected with MP12 (MOI 5) for one hour, followed by removal of viral inoculum, and addition of growth medium containing the inhibitors. At 18 hpi, supernatants were collected for standard plaque assay. The mean and SD (N = 3) are plotted. * *p*-value ≤ 0.05 vs. DMSO control; # *p*-value ≤ 0.05 when compared to same drug concentration of rapamycin alone.

**Figure 7:**
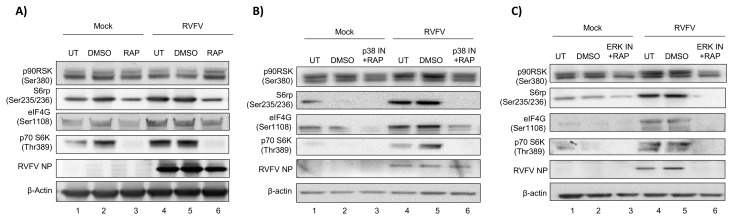
Rapamycin in combination with the p38 MAPK inhibitor SB203580 or the ERK inhibitor PD0325901 decreases translational signaling and RVFV NP production. H2.35 cells were serum starved for 72 h and then were pre-treated with either 10 µM RAP (**A**), RAP + p38 IN (**B**), or RAP +ERK IN (**C**). Cells were infected with MP12 (MOI 5) for one hour, followed by removal of viral inoculum, and addition of growth medium containing the inhibitors. At 18 hpi, cell lysates were collected for western blot analysis. UT = untreated. Images are representative of biological replicates.

In order to build on our success using the FDA approved drug rapamycin to inhibit RVFV pathogenesis, we sought to use other kinase inhibitors in combination with rapamycin. We were interested in identifying combinations of compounds that might ultimately allow animals to be completely protected from RVFV pathogenesis, as compared to the partial protection we observed with rapamycin treatment alone. Due to the relatively non-toxic nature of SB203580 and PD0325901 and the decreasing trend in viral replication noted with both inhibitors, our next step was to determine if combining them with rapamycin would result in further improvement on rapamycin’s antiviral activity. H2.35 cells were surprisingly tolerant of the drug combinations of rapamycin plus the p38 MAPK inhibitor SB203580 displaying a CC_50_ of well over 100 µM and ERK inhibitor PD0325901 plus rapamycin with a CC_50_ of over 50 µM (Figure 6A, Table 1). Robust inhibition of RVFV MP12-luc was observed with the combination treatments: ~60% inhibition at 3.125 µM, the lowest concentration tested (Figure 6B). This is a marked improvement from the inhibition observed with SB203580 (Figure 4B), PD0325901 (Figure 5B), or rapamycin alone (Figure 6B) [27]. If the effect of combining these two inhibitors had merely been additive in this case, the same dramatic decrease in replication would not have been seen. Statistical analysis of the curves confirmed that they were different than rapamycin treatment alone (*p*-value < 0.0001). Phosphorylation of S6 ribosomal protein, eIF4G, and p70 S6K and RVFV NP levels were markedly decreased with both inhibitor combinations (Figure 7A,B) and rapamycin treatment alone (Figure 7C). In addition, a significant reduction of RVFV titers was observed, even more so than that observed with rapamycin treatment alone (Figure 6C,D). These results demonstrate that the combination of rapamycin with the p38 MAPK inhibitor SB203580 or the ERK inhibitor PD0325901 resulted in robust inhibition of RVFV replication and decreased viral protein production.

## 4. Discussion

In spite of many years of research on antiviral drugs, few viruses outside of HIV, influenza, and the hepatitis viruses have effective antiviral treatments. Traditionally, antivirals have focused on targeting viral proteins and thus proteins specific to the virus itself. However, RNA viruses are inherently error-prone; they often quickly mutate in the presence of drugs that target viral proteins and viral enzymes [53,54]. Based on this, our lab is focusing on host based proteins as therapeutic targets, which will likely be harder for viruses to develop escape mutations. This approach has been shied away from in the past due to the toxic nature of drugs aimed at normal cellular processes needed for homeostasis. However, many of these types of drugs are used, often successfully, in slowing or stopping the progression of cancer. Viruses invade cells and turn on/off cellular pathways to make more progeny. Cancer cells, in a similar way, activate or inhibit cellular pathways resulting in unchecked cellular proliferation. A potential benefit to using kinase inhibitors in acute viral infections would be the short duration of use. Chemotherapy drugs used against cancer often have to be used for months at a time; use of these drugs for acute viral infections would only be needed for a few days to a week in duration to achieve a therapeutic effect. This greatly reduces the toxic effects these drugs have on our bodies. 

**Figure 8:**
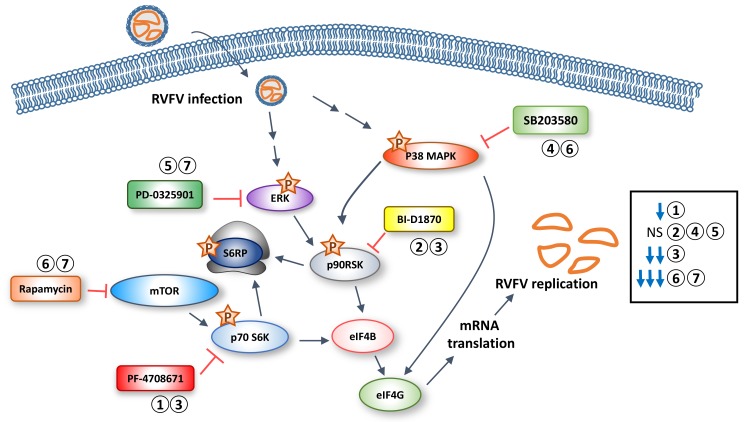
Schematic depicting the influence of kinase inhibitors on RVFV replication. RVFV infection leads to induction of ERK and p38 MAPK phosphorylation, followed by phosphorylation of p70 S6K and p90RSK. p70 S6K phosphorylation can be induced via mTOR activity, whereas p90RSK is phosphorylated by ERK and/or indirectly through p38 MAPK. Both p70 S6K and p90RSK can phosphorylate S6 ribosomal protein (S6RP) and eIF4B, leading to increased mRNA translation. The numbers above the kinase inhibitors refer to the manuscript figure number where the inhibition data are presented. The influence of the 5 different kinase inhibitors on RVFV replication tested alone or in combination is summarized on the right side of the diagram. NS = no significant change in RVFV titers.

The current study focuses on kinases in the translation pathway as a leverage point to fight RVFV (Figure 8). During our previous set of experiments, we noted the robust phosphorylation of p70 S6K and downstream proteins in this pathway, S6 ribosomal protein and eIF4G. Inhibition of p70 S6K through rapamycin treatment suppresses RVFV in vitro and prevents RVFV induced pathogenesis [27]. In the current study, we expanded our analysis to other kinases that regulate translation, including p90RSK, ERK, and p38 MAPK. We were interested in p90RSK in particular because it is also capable of phosphorylating S6 ribosomal protein, a key player in translation [55]. p90RSK is a multi-domain kinase with two non-identical, functional kinase domains consisting of a 100 amino acid linker region, and a carboxy-terminal docking site [56] with several important activation sites including Ser380, Thr359, Ser363 and Thr573 [32]. Phosphorylation of these sites follows various growth signals, serum stimulation, oncogenic perturbation, or hormonal stimuli through the action of ERK kinases [32,56,57]. When growth signals activate ERK, it docks on the C terminal docking domain and phosphorylates the activation loops of the C-terminal kinase domain (CTKD) at Thr573 followed by autophosphorylation of Ser380, located within the hydrophobic motif of the linker region. This is followed by potential phosphorylation of Thr359/Ser363 by ERK in the linker region. Once Ser380 is phosphorylated, PDK1 can dock and phosphorylate Ser221 in the N-terminal kinase domain (NTKD) leading to full activation of p90RSK [31,55,57]. Activation is followed by phosphorylation of downstream substrates such as S6 ribosomal protein and eIF4B. Interestingly, when we inhibited p90RSK through the p90RSK specific inhibitor BI-D1870, we saw a slight, but not statistically significant, increase in viral replication. These results suggest that RVFV hijacks this pathway to facilitate its replication. In addition, inhibition of p90RSK in RVFV cells did not result in a significant reduction of p90RSK (Ser380), S6 ribosomal protein or eIF4G phosphorylation, while in mock infected cells the inhibitor treatment suppressed these phosphorylation events. It is possible that p90RSK is modified in a way following RVFV infection that makes it more resistant to BI-D1870 inhibition. An alternative explanation is that the increased phosphorylation and subsequent activity of p70 S6K in RVFV infected cells compensates for any loss in p90RSK activity, resulting in minimal effect on S6 ribosomal and eIF4G phosphorylation. 

Previous work by Popova et al. demonstrated that inhibition of p38 MAPK caused a significant increase in RVFV replication, and inhibition of ERK kinase caused a decrease in RVFV replication [26]. These studies are only in partial agreement with results presented within, as we observed a moderate decrease in RVFV replication in the presence of both p38 MAPK and ERK inhibitors. Human small airway epithelial cells (HSAECs) were used in the previous study, whereas in this study these pathways were studied in mouse hepatocytes, which may explain the observed differences. In addition, our testing of ERK and p38 MAPRK inhibitors also included the use of MP12-Luc which lacks the viral protein NSs. However, our data demonstrated similar reduction of RVFV replication with the ERK and p38 MAPK inhibitors in the presence or absence of NSs. These results suggest that NSs has no influence on ERK and p38 MAPK functions. Both of the kinases are upstream of p90RSK and induce phosphorylation of p90RSK in response to different stimuli [30,31,49,55]. ERK is often active in a cellular environment where growth and differentiation are occurring, whereas p38 MAPK is activated in times of cellular stress and results in a cellular environment leading to apoptosis [30,31,49,55]. Phosphorylation of p90RSK has been observed following severe acute respiratory syndrome (SARS) coronavirus infection [30]. In contrast to what is observed in non-infected cells, phosphorylation of Ser380 in response to SARS coronavirus infection was not dependent on phosphorylation of Thr573 [30], an event which is important in ERK stimulated p90RSK activation [58]. Rather phosphorylation of p90RSK on Ser380 was dependent on p38 MAPK activity, as treatment with the p38 MAPK inhibitor SB203580 prevented this phosphorylation event [30]. p90RSK Ser380 phosphorylation in RVFV infected cells was more sensitive to ERK inhibition than p38 MAPK inhibition, suggesting that p90RSK phosphorylation events follow the classical form of regulation. Inhibition of ERK and p38 MAPK in RVFV infected cells did not result in a significant inhibition of S6 ribosomal protein or eIF4G, as compared to p70 S6 kinase inhibition. These data suggest that inhibiting ERK and p38 MAPK has minimal effect on translation, possibly due to a large redundancy in this pathway. 

An important component of the current study is the combined use of inhibitors to suppress viral replication. When cells were treated with the ERK inhibitor PD0325901 or the p38 MAPK inhibitor SB203580 together with rapamycin, an FDA approved drug with demonstrated efficacy against RVFV [27], a significant decrease in viral replication was observed (Figure 6, Figure 7 and Figure 8). Likewise a significant decrease in viral replication was seen when cells were treated with both the p70 S6K inhibitor PF-4708671 and the p90RSK inhibitor BI-D1870 (Figure 3 and Figure 8). In all cases, the combination of inhibitors resulted in the largest suppression of viral replication as compared to any inhibitor used in isolation. They also resulted in the largest suppression of translational signaling, suggesting that more potent shutdown of this signaling pathway is needed to suppress RVFV replication. These data indicate that targeting multiple key kinases within the translational pathway allows for the most robust viral inhibition.

A caveat of our study is that we analyzed viral replication using a reporter virus lacking the viral protein NSs (RVFV MP12 ∆NSs-Luc) in addition to wildtype RVFV MP12 containing NSs. NSs is the main RVFV virulence factor, responsible for suppression of interferon, through multiple mechanisms including inducing degradation of PKR and TFIIH p62 [59,60]. Therefore it would be expected that greater viral inhibition following inhibitor treatments may be observed in the absence of NSs when cells are able to mount a more effective antiviral response. Indeed we observed greater inhibition of RVFV replication in the absence of NSs with p70 S6K inhibitor PF-4708671 treatment as compared to in the presence of NSs (Figure 1). However, all other inhibitor treatments either alone or in combination displayed similar inhibition in the presence or absence of NS. 

Given the importance of the translational pathway, it would be of interest to determine the sensitivity of other viruses to these kinase inhibitors. This analysis would enable a greater understanding of the kinase requirements for viruses from different families. While we think inhibition of translation is at least in part responsible for the positive antiviral effects of these drugs, we fully acknowledge this is most likely only part of the story. For example, ERK and p38 MAPK are involved with many cellular events inducing cell cycle regulation, cell survival, transcription, and innate immune responses [61,62,63]. As such, future work focused on the molecular mechanisms behind the beneficial aspects of these drugs is needed. 

## 5. Conclusions

One of our overarching goals is to find safe, effective, antiviral drugs, and, to this end, we are currently focused on host-based therapeutics. Worldwide, effective antiviral therapies to date have been few, so we looked at this problem anew in the hope of finding novel ways to fight deadly viral infections. Our current strategy is to inhibit machinery needed for viruses to propagate, and, in this case, that deprivation is in the form of translational shutoff through kinase inhibitors. This study establishes that combination therapy can not only be effective at slowing viral replication, but that it can be done with lower drug concentrations than what is necessary for individual drug therapy.

## Figures and Tables

**Table 1:** Summary of kinase inhibitors used and their activities against Rift Valley fever virus (RVFV).

Kinase Inhibitor	Target	CC_50_ (µM)	EC_50_ (µM)	SI ^†^
PF-4708671	p70 S6K	>50 *	17	>2.9
BI-D1870	p90RSK	>100 *	N.A. ^#^	ND ^$^
PF-4708671 + BI-D1870	p70 S6K + p90RSK	62	<1.56 *	>40
SB203580	p38 MAPK	>100 *	>100 *	ND
PD0325901	ERK	>100 *	>50 *	ND
Rapamycin	mTOR/p70 S6K	>100 *	18	>5.6
Rapamycin + SB203580	mTOR/p70 S6K + p38 MAPK	>100 *	<1.56 *	>64
Rapamycin + PD0325901	mTOR/p70 S6K + ERK	>50 *	<1.56 *	>32

**^†^** SI = CC_50_/EC_50_. This value is estimated when the CC_50_ or EC_50_ is not definitively determined; * A definitive value could not be determined due to the lack of a defined top or bottom plateau; ^#^ Enhancement of viral replication was observed; ^$^ Not determined.
